# Supplementary material for: The toposiomerase IIIalpha-RMI1-RMI2 complex orients human Bloom’s syndrome helicase for efficient disruption of D-loops
Source: Nat Commun. 2022 Feb 3;13:654. doi: 10.1038/s41467-022-28208-9 (PMC8813930; doi:10.1038/s41467-022-28208-9)
Supplement: Supplementary file 1 — Supplementary Information [file 41467_2022_28208_MOESM1_ESM.pdf]

SUPPLEMENTARY INFORMATION

The toposiomerase IIIalpha-RMI1-RMI2 complex orients human Bloom's syndrome helicase for efficient disruption of D-loops

Gábor M. Harami*, János Pálinkás*, Yeonee Seol, Zoltán J. Kovács, Máté Gyimesi, Hajnalka Harami-Papp, Keir C. Neuman, and Mihály Kovács

SUPPLEMENTARY FIGURES

Figure S1

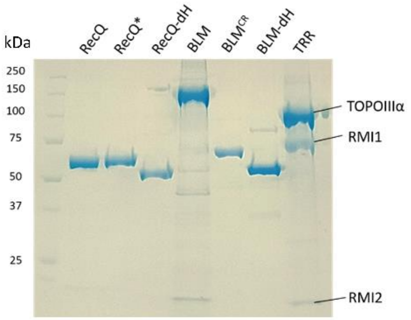

Supplementary Fig. 1. SDS-PAGE analysis of protein constructs used in the study
Proteins were separated on a 4-20% SDS-PAGE and stained with Coomassie brilliant blue. The experiment shown was performed to represent the apparent molecular sizes of used constructs in a single gel. Purity of individual preparations was routinely checked separately as part of the purification protocol and showed reproducible results. Source data are provided as a Source Data file.

Figure S2

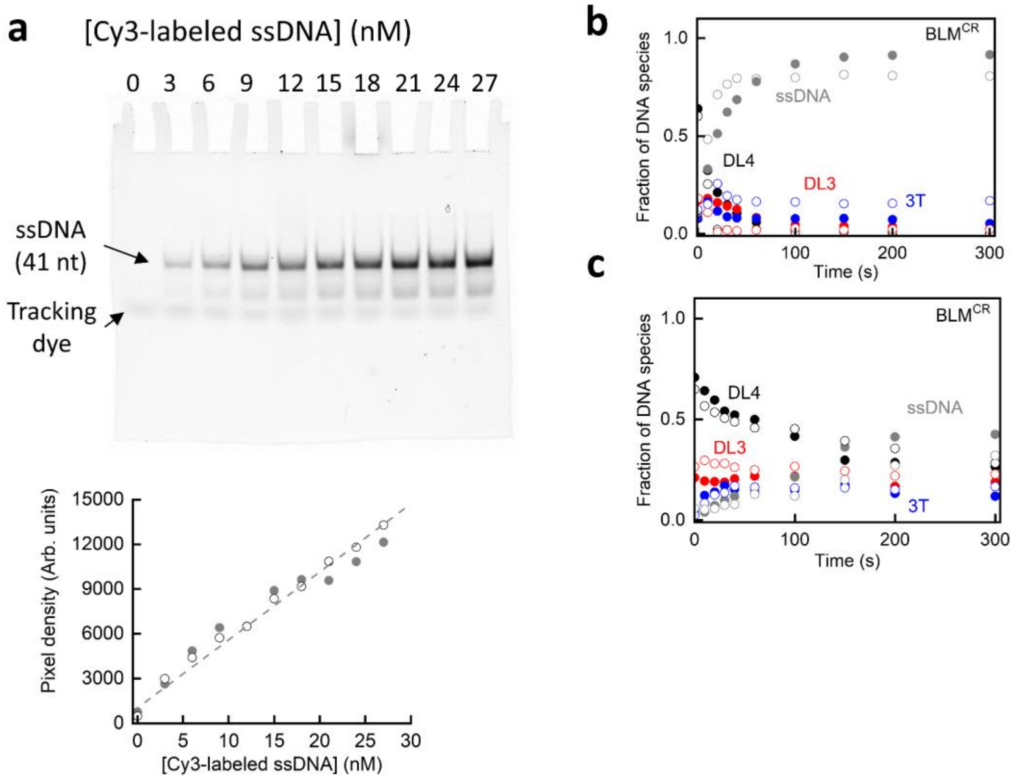

Supplementary Fig. 2. Controls for I-trap D-loop disruption experiments
(**a**) Example electrophoretogram of various concentrations of Cy3-labeled ssDNA (D3 (41 nt), invading strand of the D-loop structure; see Methods for oligonucleotide sequences) run on PAGE. The experiment was done twice and showed reproducible results. Experimental conditions were as described for D-loop processing experiments (see Methods). 10 µl labeled ssDNA with the indicated concentration plus 4 µl loading dye was loaded onto the gel per sample. The background subtracted fluorescence pixel density of bands detected after electrophoresis increased linearly with increasing ssDNA concentration in the investigated regime (data for 2 experiments are shown, dashed line shows

linear fit to the data (slope: 455 ± 21 nM^{-1}; intercept: 1040 ± 254)). These control experiments confirm that the detection method and the D-loop construct concentration applied in the kinetic experiments (30 nM) are suitable for precise quantification required for kinetic analysis.

(**b**) Kinetic profiles of two control experiments monitoring DL4 unwinding by BLM^{CR} in the absence of ssDNA trap strand. After stopping the reaction, 3 µM I-trap strand was added to the mixture to prevent reannealing of unwound DNA molecules. As expected, the omission of the ssDNA trap strand during the reaction markedly accelerated the decomposition of all multi-stranded DNA species (cf. Fig. 2b).

(**c**) Kinetic profiles of two control experiments monitoring DL4 unwinding by BLM in the presence of 15 µM trap strand (5 times higher than in Fig. 2c). Elevation of trap strand concentration slightly reduced the amplitude of the first phase of DL4 decomposition, similar to that previously observed for RecQ[1], suggesting an increase in the fraction of the non-productive unwinding runs (DLN, Fig. 3).

DNA species are color coded as in Fig. 3**.** Experiment sets 1 and 2 are shown as solid and open circles, respectively. DNA species are described in Fig. 2b and in Methods. Source data are provided as a Source Data file.

Figure S3

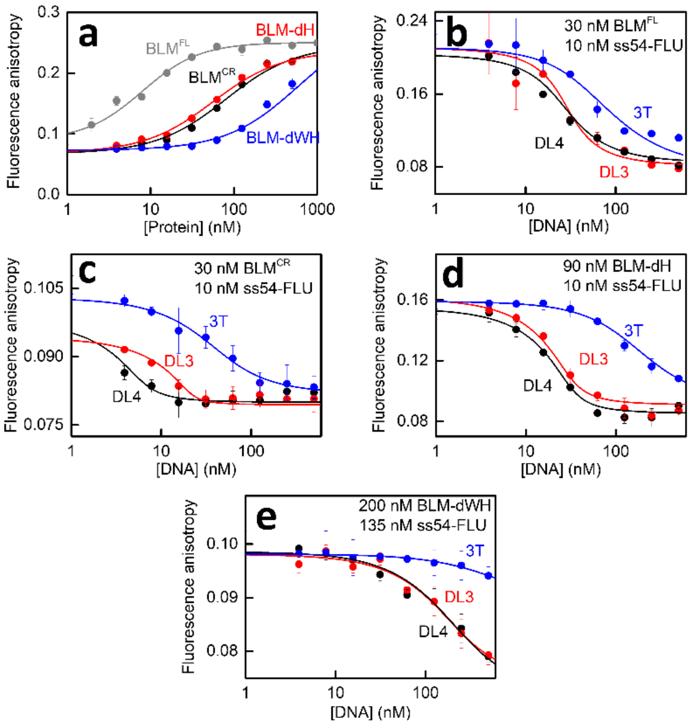

Supplementary Fig. 3. Binding of BLM protein constructs (Fig. 1c) to ssDNA and DNA species involved in D-loop processing experiments

(**a**) Fluorescence anisotropy titration of 10 nM fluorescein-labeled ss54-FLU DNA substrate (54-mer ssDNA molecule, see Methods) with increasing amounts of BLM constructs in the absence of ATP. Solid lines show hyperbolic fits. Deletion of the WH domain markedly reduces the ssDNA binding affinity of BLM. Determined K_d values are listed in Supplementary Table 1. Means ± SEM for n = 3 replicates are shown.

(**b-e**) Competitive fluorescence anisotropy titrations using increasing concentrations of 3T (blue), DL3 (red) or DL4 (black) DNA substrates to compete with ss54-FLU for binding to (**b**) BLM^{FL}, (**c**) BLM^{CR}, (**d**) BLM-dH and (e) BLM-dWH constructs. ss54-FLU signal was monitored. The concentrations of helicase constructs and ss54-FLU were kept constant at the values shown in the panels. Means ± SEM for n = 3 replicates are shown. Solid lines are best fits based on Supplementary Equations 1-2. Determined K_d values are listed in Supplementary Table 1.

The observed strong binding to the DL4, DL3 and 3T species by all investigated, WHD-containing BLM constructs (i.e. all constructs except BLM-dWH) confirmed that practically all DNA molecules were initially enzyme-bound in the unwinding experiments. Furthermore, the rebinding rate constants (k_R for DL4 and DL3, k_R' for 3T) determined from global fits shown in Figs. 4a, 5, 7 were in the same range for all BLM helicase constructs, further suggesting that binding to these DNA structures is not markedly influenced by the deletion of the NC regions and the HRDC domain of BLM (Supplementary Tables 1-2). Source data are provided as a Source Data file.

Figure S4

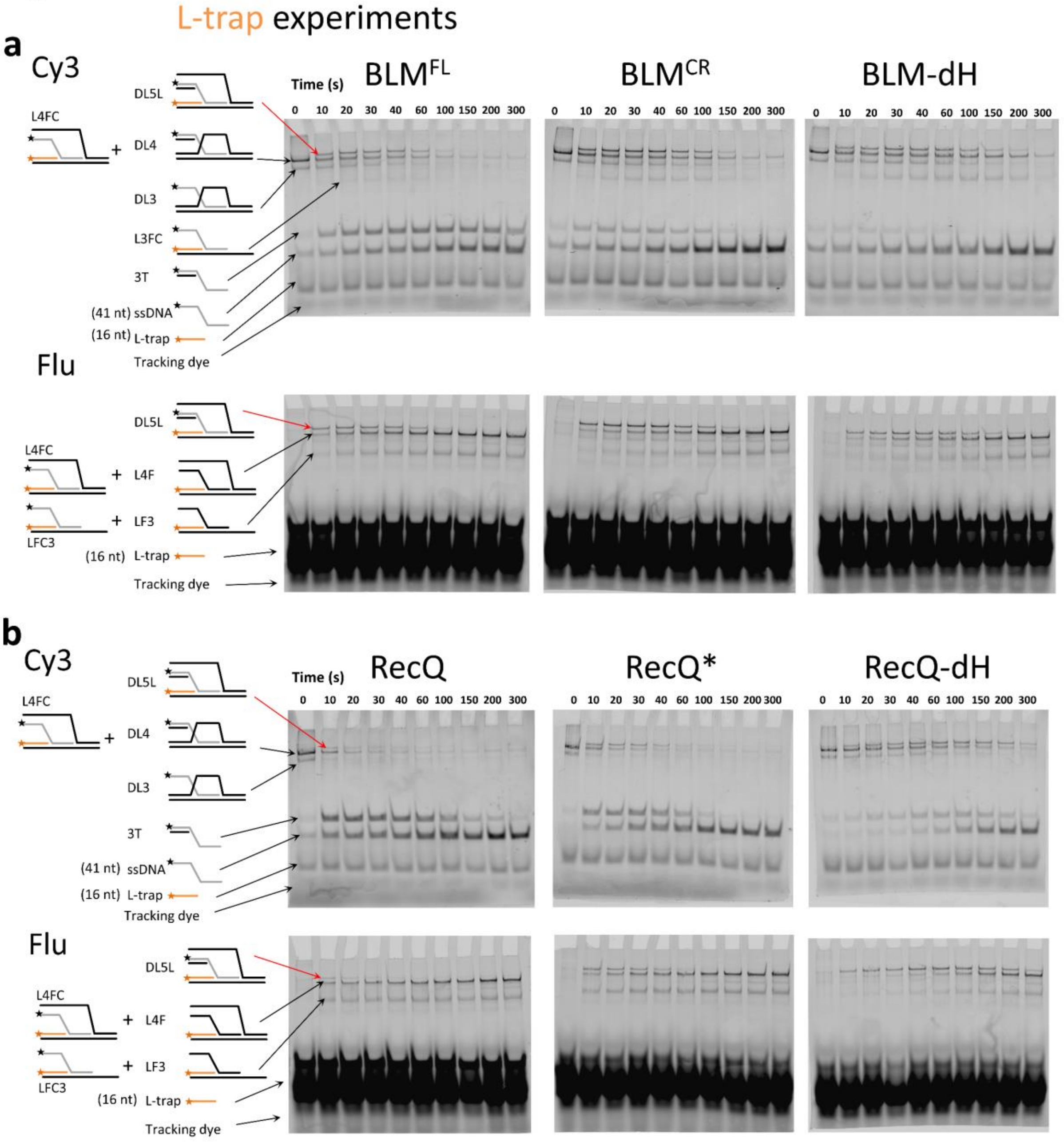

Supplementary Fig. 4. Representative electrophoretograms of L-trap D-loop unwinding experiments monitoring Cy3 or fluorescein signals

Representative electrophoretograms of D-loop unwinding experiments using (a) BLM and (b) RecQ constructs (Fig. 1c) in the presence of L-trap (100 nM enzyme, 30 nM DL4, 1.5 µM I-trap (unlabeled ssDNA trap strand) and 1.5 µM L-trap). Experiments were performed as in Fig. 5a. Cy3 and fluorescein (Flu) signals were detected separately for each gel. In the L-trap experiments, new bands appeared in

addition to the ones previously observed in I-trap only experiments (DL4, DL3, 3T, ssDNA). DNA structures corresponding to different bands were identified based on the results of Supplementary Fig. 6a. A band migrating slower than DL4 was observed monitoring the Cy3 or fluorescein signals. This band was assigned to the DL5L DNA structure (Supplementary Fig. 6a), originating from unwinding the leftward dsDNA arm of DL4 (Fig. 5a, Supplementary Fig. 7).

Additional bands are identified as the four stranded L4F (shows fluorescein signal only) and L4FC (fluorescein and Cy3 signal) and as the three-stranded LF3 (shows fluorescein signal only) and LFC3 (fluorescein and Cy3 signal) DNA structures (Supplementary Fig. 6a). Considering possible D-loop binding orientations, L4FC is a product of DL3 (initially present in the DL4 preparation and bound by BLM) processing occurring via the DLL pathway (Supplementary Fig. 7, DL3 – DLL pathway). In addition, L4FC can also arise from the DL5L structure (Supplementary Fig. 7, PE pathway) due to enzyme rebinding. Similarly, L4F, LF3 and LFC3 will form due to enzyme rebinding and subsequent processing of intermediate DNA structures, ultimately yielding the final reaction products L4F, LF3 (fluorescein signal only) and ssDNA (Cy3 signal only). For all constructs, three independent experiments were performed and showed reproducible kinetic profiles. Densitometry of DNA species DL4, DL3, 3T and DL5L obtained from the Cy3 signal and DL5L obtained from the fluorescein signal are shown in Figs. 6a and 7a. Densitometry of DNA species LF3 + LFC3, L4F + L4FC, detectable via the fluorescein signal, is shown in Supplementary Fig. 9. Source data are provided as a Source Data file.

Figure S5

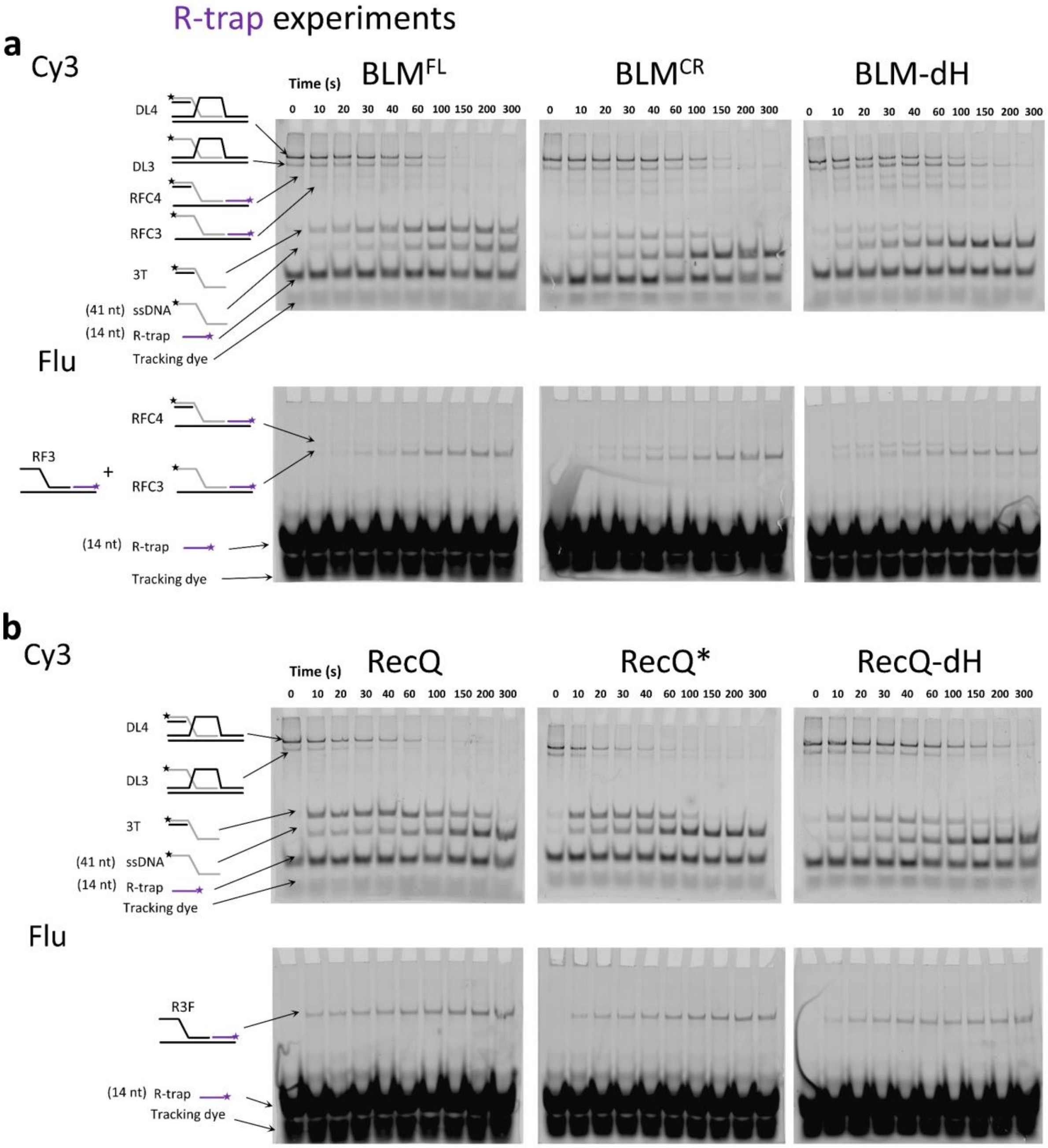

Supplementary Fig. 5. Representative electrophoretograms of R-trap D-loop unwinding experiments monitoring Cy3 or fluorescein signals

Representative electrophoretograms of D-loop unwinding experiments using (**a**) BLM and (**b**) RecQ constructs (Fig. 1c) in the presence of R-trap (100 nM enzyme, 30 nM DL4, 1.5 µM I-trap (unlabeled ssDNA trap strand) and 1.5 µM R-trap). Experiments were performed as in Fig. 5b. Cy3 and fluorescein (Flu) signals were detected separately for each gel. In the R-trap experiments, new bands appeared in addition to the ones previously observed in I-trap only experiments (DL4, DL3, 3T, ssDNA). DNA structures corresponding to different bands were identified based on the results of Supplementary Fig. 6b. Importantly, a band migrating slower than DL4 was not observed for any of the investigated helicase constructs. Based on Fig. 5a and Supplementary Fig. 6b, unwinding of the rightward dsDNA arm of the DL4 structure should lead to the formation of the DL5R structure, migrating slower than DL4 (Supplementary Fig. 6b). Lack of this structure indicates that the investigated helicases do not

efficiently catalyze the initial unwinding of the rightward dsDNA arm and/or the amount of the formed products is below of our detection limit.

Additional bands are identified as the four-stranded RFC4 (fluorescein and Cy3 signal) and the three-stranded RFC3 (fluorescein and Cy3 signal) and RF3 (fluorescein signal only) structures. These structures will form due to enzyme rebinding and subsequent processing of intermediate DNA structures. For all constructs, three independent experiments were performed and showed reproducible kinetic profiles. Densitometry of DNA species DL4, DL3, 3T obtained from the Cy3 signal are shown in Figs. 6b, 7b. Densitometry of DNA species RFC4, R3F and RFC3, detectable via the fluorescein signal, is shown in Supplementary Fig. 9. Source data are provided as a Source Data file.

Figure S6

a L-trap containing DNA structures

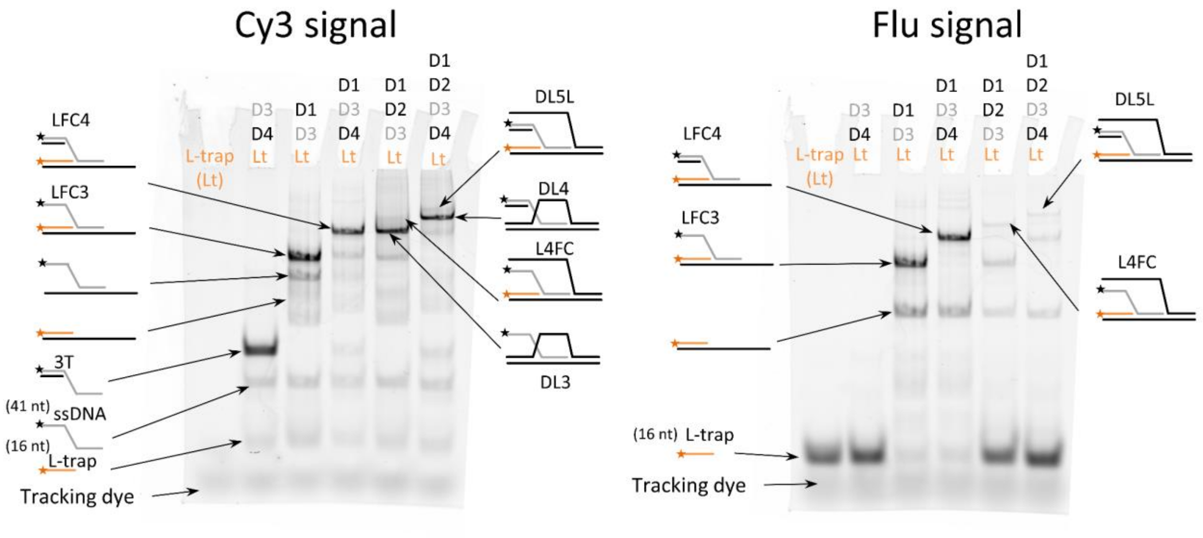

b R-trap containing DNA structures

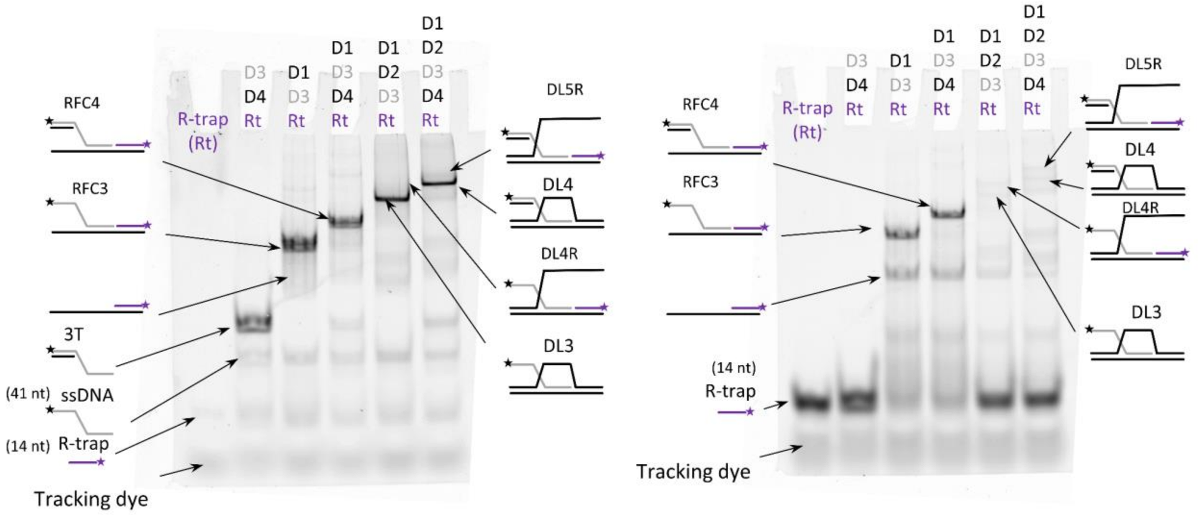

Supplementary Fig. 6. Identification of DNA species observed during L-trap and R-trap D-loop unwinding experiments

Shown are Cy3 and fluorescein (Flu) fluorometric images of a 12 % native PAGE electrophoretogram of DNA species involved in DL4 disruption assays performed in the presence of fluorescein labeled (**a**) L-trap or (**b**) R-trap DNA strands (Fig. 4; see also the model shown in Supplementary Fig. 7, oligonucleotides are described in Methods). DNA structures were generated by mixing and annealing the indicated oligonucleotides (final concentration: 30 nM each; note that in L-trap and R-trap experiments 1.5 µM was used). The relative mobility of bands compared to that of the tracking dye was used to identify DNA structures appearing in the D-loop unwinding experiments (Supplementary Figs. 4-5, 8, 11-12, 14). L- and R-trap experiments were performed independently, as results were in line with the predictable DNA structures based on the complementarity of DNA strands and with previous results (Fig. 2b). Source data are provided as a Source Data file.

Figure S7

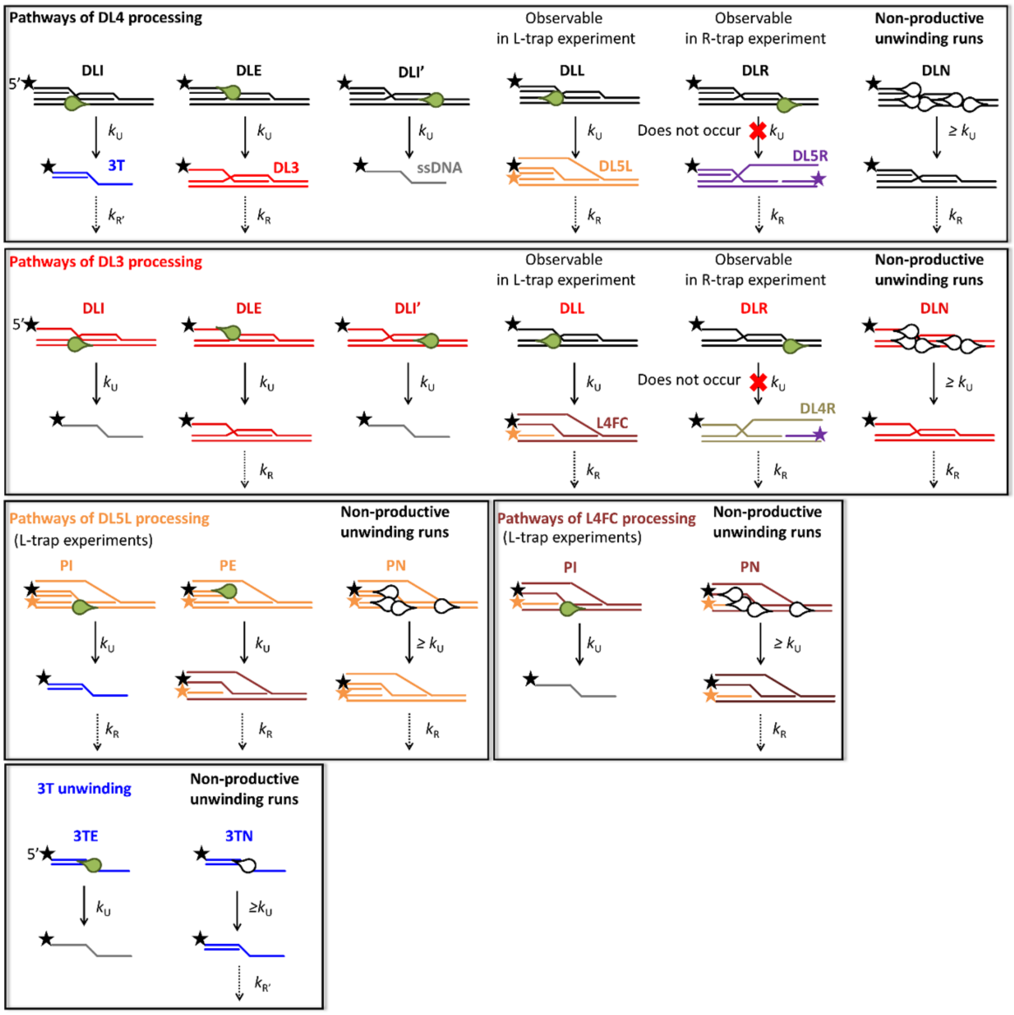

Supplementary Fig. 7. Pathways of processing major observable DNA species initially present or generated during the L-trap or R-trap experiments

In the new D-loop experiments, in addition to the pathways shown in Fig. 3, the DLL and DLR pathways can be independently resolved with the addition of L-trap or R-trap (Fig. 5). Unwinding of DL4 and DL3 in the DLL pathway will lead to the formation (with rate constant k_U) of the DL5L (orange) and L4FC (brown) DNA structures, respectively, which are observable in L-trap experiments (Supplementary Figs. 4, 6a). k_U represents a lower bound for non-productive events and also for the unwinding rates as reaction kinetics are not resolved before the first 10 s. In the DLR pathway, unwinding of DL4 and DL3 (with rate constant k_U) will lead to the formation of the DL5R (purple) and DL4R (olive) structures, respectively. These structures, if present, would be observed in R-trap experiments (Supplementary Figs. 5, 6b). Importantly, our results (Supplementary Fig. 5) indicate that none of the investigated helicase constructs uses the DLR pathway. Unwinding of the invading strand in the DL5L or L4FC structures (PI pathways) will lead to formation of 3T and ssDNA, respectively. A helicase binding orientation that leads to the unwinding of the DL5L structure outward of the invading strand, using it as the tracking strand, will lead to the formation of the L4FC structure. The PN pathway contains all unwinding events that are non-productive (due to premature termination of unwinding starting from any binding orientation or due to reannealing of the unwound dsDNA arm upstream of the strand invasion in L-trap experiments). Slow rebinding of enzyme to DNA products (inhibited by excess ssDNA trap strands, occurring with rate constant k_R) and subsequent unwinding leads to further processing of the given structures (these processes are exemplified by the disappearance of 3T or DL5L after their accumulation). Pathways involving DL5R and DL4R are not shown in the model as the DLR pathway

was not observed. The model was used in global fits to results shown in Figs. 6-8, Supplementary Fig. 13.

The black star indicates the Cy3 label in the invading strand, whereas the orange and purple stars/strands represent L-trap and R-trap (with fluorescein label), respectively.

Figure S8

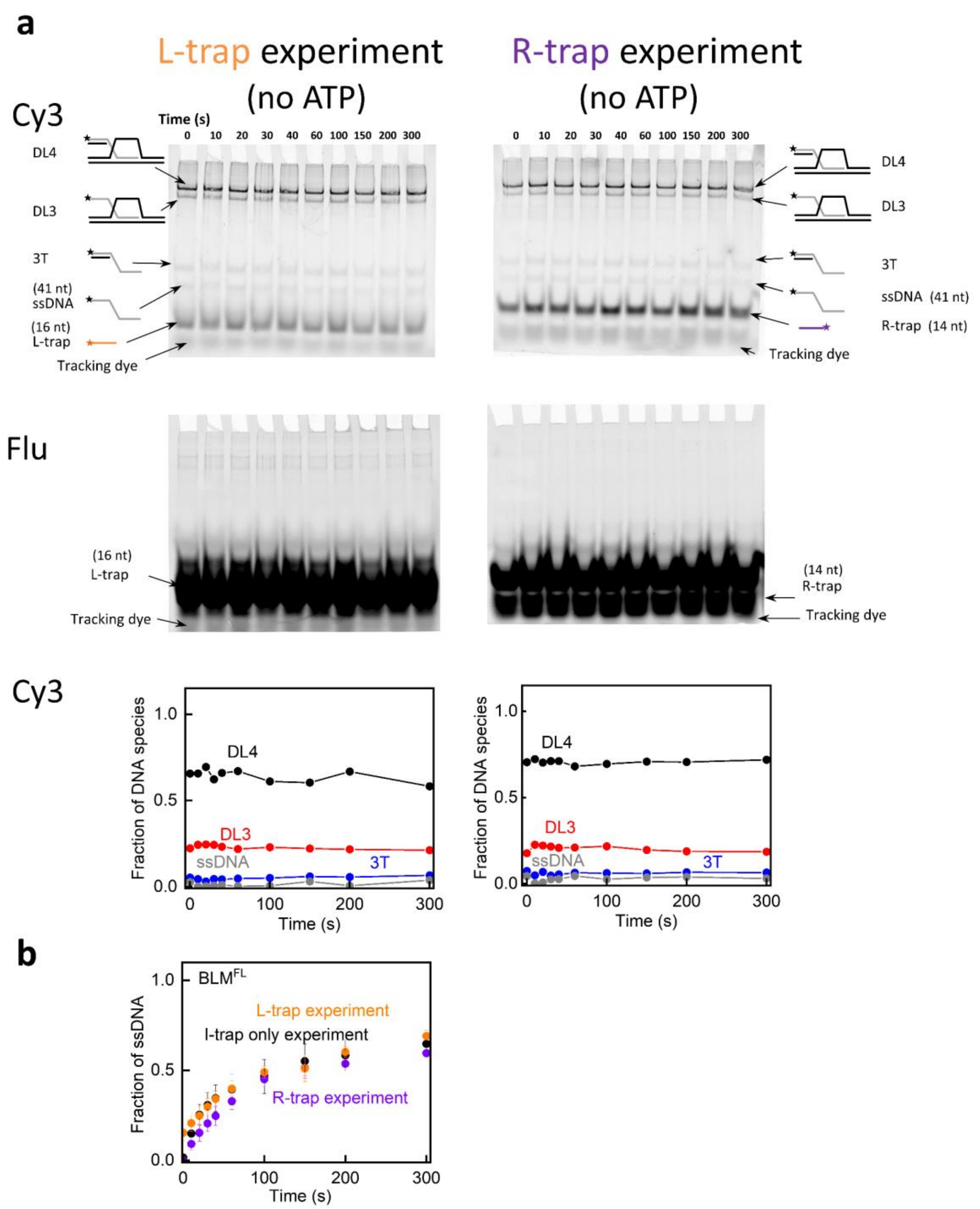

Supplementary Fig. 8. Controls for L-trap and R-trap experiments

(**a**) Shown are Cy3 and fluorescein (Flu) electrophoretograms of control D-loop unwinding experiments performed using BLM^{FL} in the absence of ATP, in the presence of L-trap or R-trap. In the absence of helicase activity, the fraction of DNA species originally present in the DL4 preparations remained constant over time, indicating that the presence of L-trap or R-trap alone does not facilitate conversion

of these DNA structures. Each experiment was performed in a single run, as they were consistent with each other and with previous findings that ATP is required for BLM-mediated unwinding[2].
(**b**) Comparison of ssDNA accumulation kinetics during DL4 processing by BLM^{FL} in the presence of L-trap (orange) or R-trap (magenta), or I-trap (black) only. Data were taken from Figs. 4a, 6a-b; means ± SEM are shown for 3 independent experiments. The similar kinetics show that the type of trap does not overall influence the kinetics of the reaction, and all data can be used in global fits using the model shown in Supplementary Fig. 7. Source data are provided as a Source Data file.

Figure S9

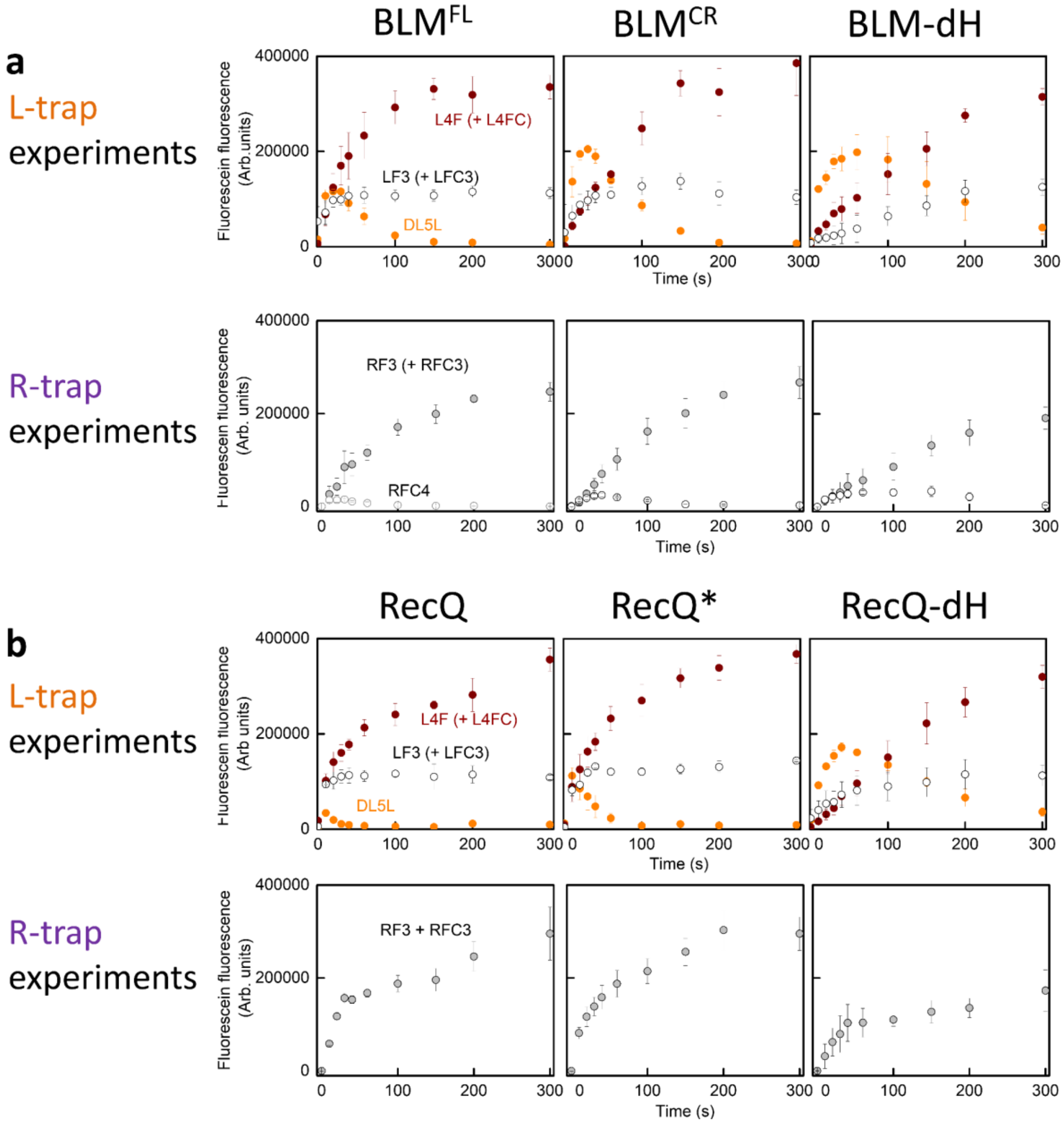

Supplementary Fig. 9. Band intensities for DNA species observed via the fluorescein signal
(**a-b**) Band intensities of DL5L, L4F, L4FC, RF3, RFC3, RFC4 from measurements of fluorescein fluorescence in the gel for L-trap and R-trap experiments (Supplementary Figs. 4-5) performed with (**a**) BLM or (**b**) RecQ constructs. DNA species are colored as in Supplementary Fig. 7, except for RF3 and RFC3. Means ± SEM are shown for 3 independent experiments with the indicated constructs. Source data are provided as a Source Data file.

Figure S10

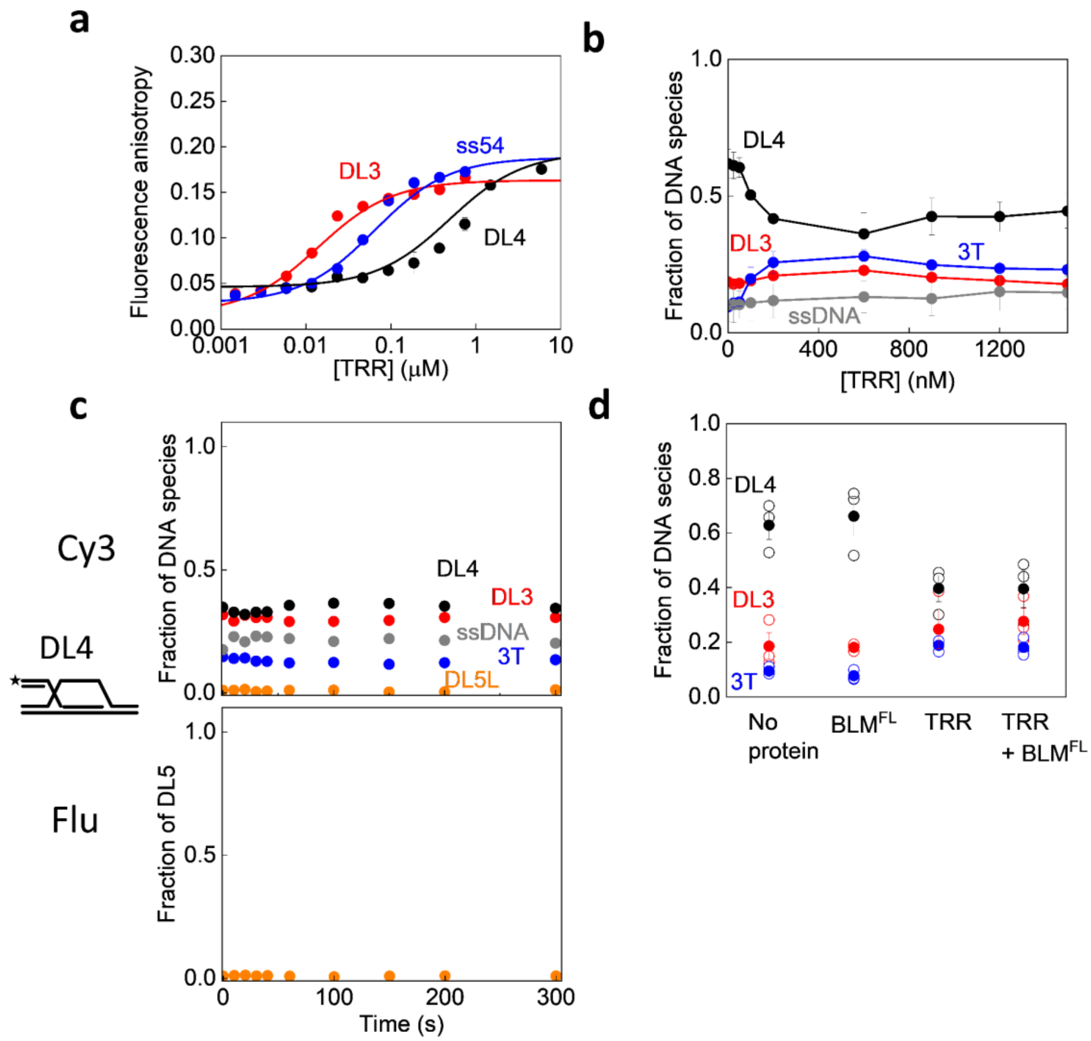

Supplementary Fig. 10. DNA binding by TRR and the effect of TRR on DNA species

(**a**) Fluorescence anisotropy measurements performed by mixing 10 nM of the indicated, fluorescein-labeled DNA species (DL4, DL3, ss54; Methods) with increasing amounts of TRR. The increase in anisotropy indicates binding of TRR to the DNA molecules. Solid lines show bets fits using a hyperbolic binding equation to determine K_d values. Binding was the strongest for DL3 (K_d = 0.013 ± 0.001 μM) followed by ss54 ssDNA (K_d = 0.064 ± 0.013 μM) and DL4 (K_d = 0.488 ± 0.07 μM). Means ± SEM are shown for n = 3 repetitions using one preparation.

(**b**) Effect of TRR on the stability of DL4, DL3 and 3T DNA species. A preparation of DL4 (which contained a fraction of DL3 and 3T) was mixed with increasing concentrations of TRR and was incubated on 37°C for 5 min. After addition of the stop solution used in D-loop unwinding experiments, DNA species were separated and visualized as in D-loop processing experiments (cf. Fig 2c). TRR decreased (but did not completely abolish) the fraction of DL4 in a concentration-dependent manner, leading to the formation of 3T. The fractions of DL3 and ssDNA did not change during the experiment, indicating that binding of TRR to DL4 partially disrupts the strand invasion, but only when the 5′ end of the strand invasion is in the double-stranded form.

(**c**) Changes in fractions of DNA species present in DL4 preparations were followed in time after addition of ATP (1 mM final) to a preincubated (5 min 37°C) mixture of TRR and the DL4 preparation. The final concentration of TRR was 1.2 μM after addition of ATP. Reactions were set up and analysis was performed as for the experiments monitoring the activity of helicases (cf. Fig. 2c). DL4 fractions were lower, while 3T fractions were higher than that observed without TRR (Fig. 2c, Supplementary Fig. 8a), as expected based on panel b. The fractions of different DNA species remained constant during

the investigated time regime, highlighting that the passive DL4 disruption activity of TRR reached an equilibrium during the preincubation period. Importantly, these results indicate that the D-loop processing measurements (Fig. 8, Supplementary Fig. 11) can be performed in the presence of the applied high TRR concentrations. The experiment was performed in a single run, as the observations were in line with the results shown on panel b, i.e. the initial fractions of DNA species were similar to those obtained in panel b with 1.2 µM TRR after 5 minutes.

(d) Fractions of DL4, DL3 and 3T DNA species after 3 min incubation of the DL4 D-loop sample with 100 nM BLM^{FL} alone, 1.2 µm TRR alone or 1.2 µm TRR plus 100 nM BLM^{FL}; in the absence of ATP. These experiments reflect the activity of proteins in the absence of ATP during the incubation period of D-loop experiments. The results are in line with fractionation change of the investigated DNA species at the 0 time point of D-loop disruption experiments performed in the presence of TRR and other helicase constructs. Individual data points (open circles, DL4: black, DL3: red, 3T: blue) and means (solid circles) ± SEM are shown for 3 independent experiments. Based on two-tailed T-test analysis, BLM alone does not change the fractionation of DNA species significantly compared to the no protein control at the 0.05 significance level, in line with the results of Supplementary Fig. 8a. In line with panel b, TRR alone significantly decreased the fraction of DL4 ($p = 0.03$) and significantly increased the fraction of 3T ($p = 0.003$) compared to the no protein control, whereas the moderate increase in the DL3 fraction is statistically not significant. Importantly, TRR + BLM behaved similar to TRR alone; the small differences between fractionation of DNA species with TRR or TRR + BLM were found to be statistically not significant.

These control experiments, together with panel b, indicate that in experiments where TRR and BLM are incubated with the D-loop structure in the absence of ATP for 3 min, TRR-facilitated D-loop disruption reaches an equilibrium and this is not affected by the presence of BLM. Source data are provided as a Source Data file.

Figure S11

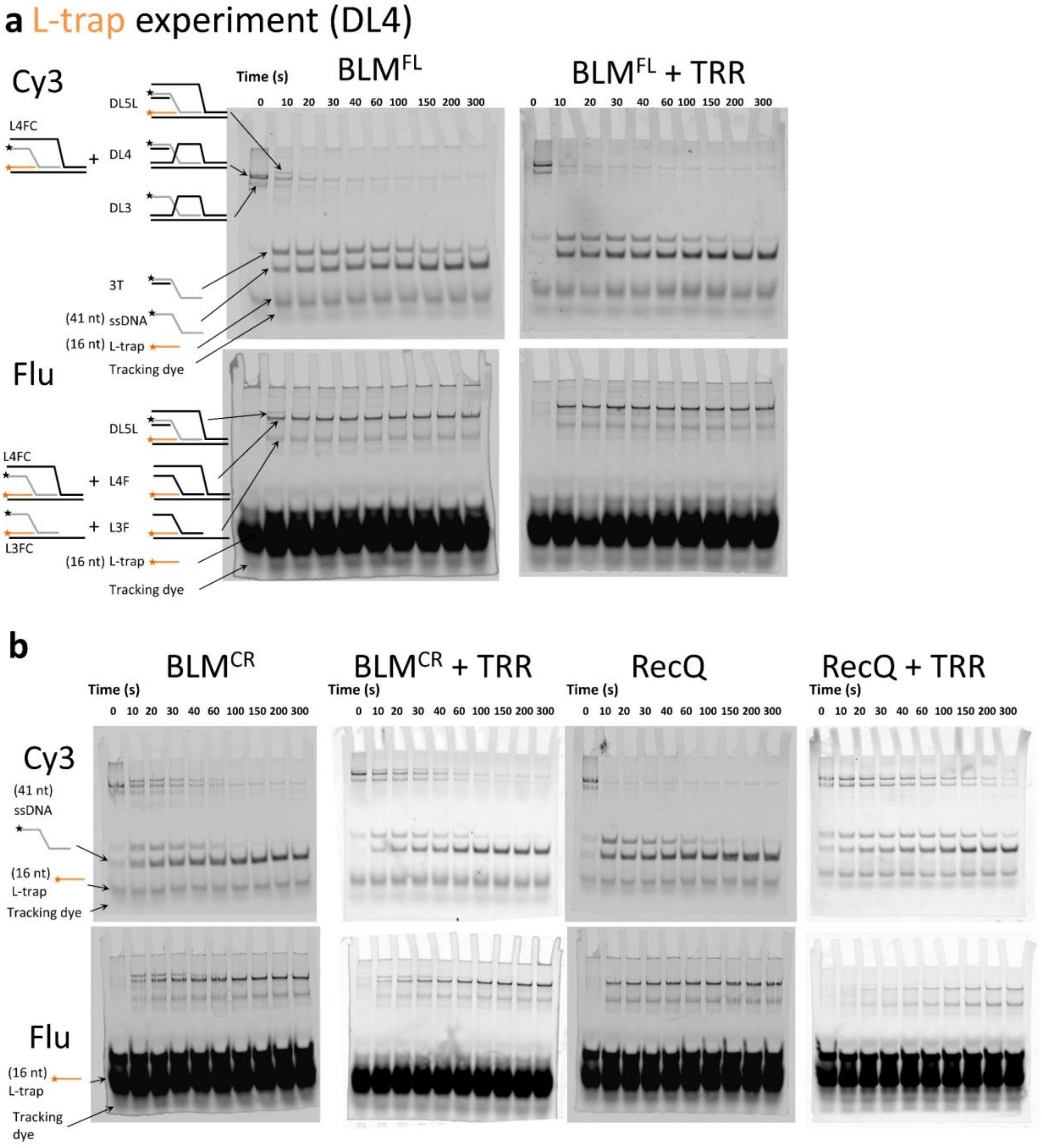

Supplementary Fig. 11. Representative electrophoretograms of L-trap D-loop unwinding experiments with and without TRR, monitoring Cy3 and fluorescein signals

(**a**) D-loop processing measurements of BLM^{FL} were done as in Fig. 5 and Supplementary Fig. 5 with or without 1.2 µM TRR. DNA species were annotated according to Supplementary Fig. 6. The presence of TRR inhibits the formation of DL4L molecule, indicating the inhibition of the DLL pathway.

(**b**) Representative Cy3 and fluorescein (Flu) electrophoretograms obtained with the indicated helicase constructs with or without 1.2 µM TRR.

Average fractions of DNA species determined from multiple experiments are shown in Fig. 8 and Supplementary Fig. 13. Experiments were performed 3 times independently and showed identical patterns for DNA species. Source data are provided as a Source Data file.

Figure S12

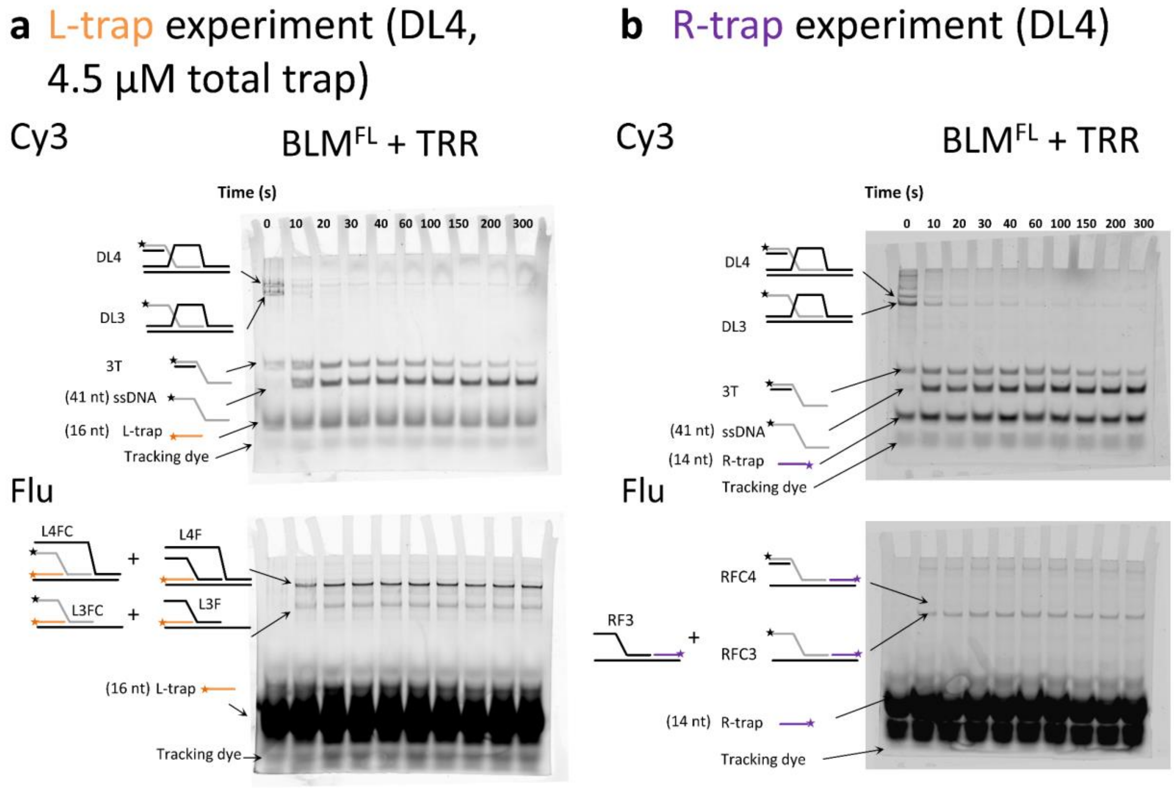

Supplementary Fig. 12. DL4 L-trap experiment of BLM^{FL} + TRR in the presence of increased trap stand concentration and DL4 R-trap experiment with BLM^{FL} + TRR

(**a**) Experiments performed as in Fig. 5 and Supplementary Fig. S11 but, instead of a 3 µM total trap stand concentration, 4.5 µM was used (2.25 µM of each of I-trap and L-trap). These experiments confirmed that the absence of the DLL pathway is not caused by depletion of trap strand by TRR.

(**b**) R-trap experiments performed as in Supplementary Fig. S6 in the presence of 1.2 µM TRR. The absence of the DL4R DNA species (Fig. 5) with or without TRR (Supplementary Fig. 6) indicates that TRR does not increase the preference of BLM for the DLR pathway (for pathways see Supplementary Fig. 7). Results were in line with previous independent results and each experiment was therefore performed in a single run.

Figure S13

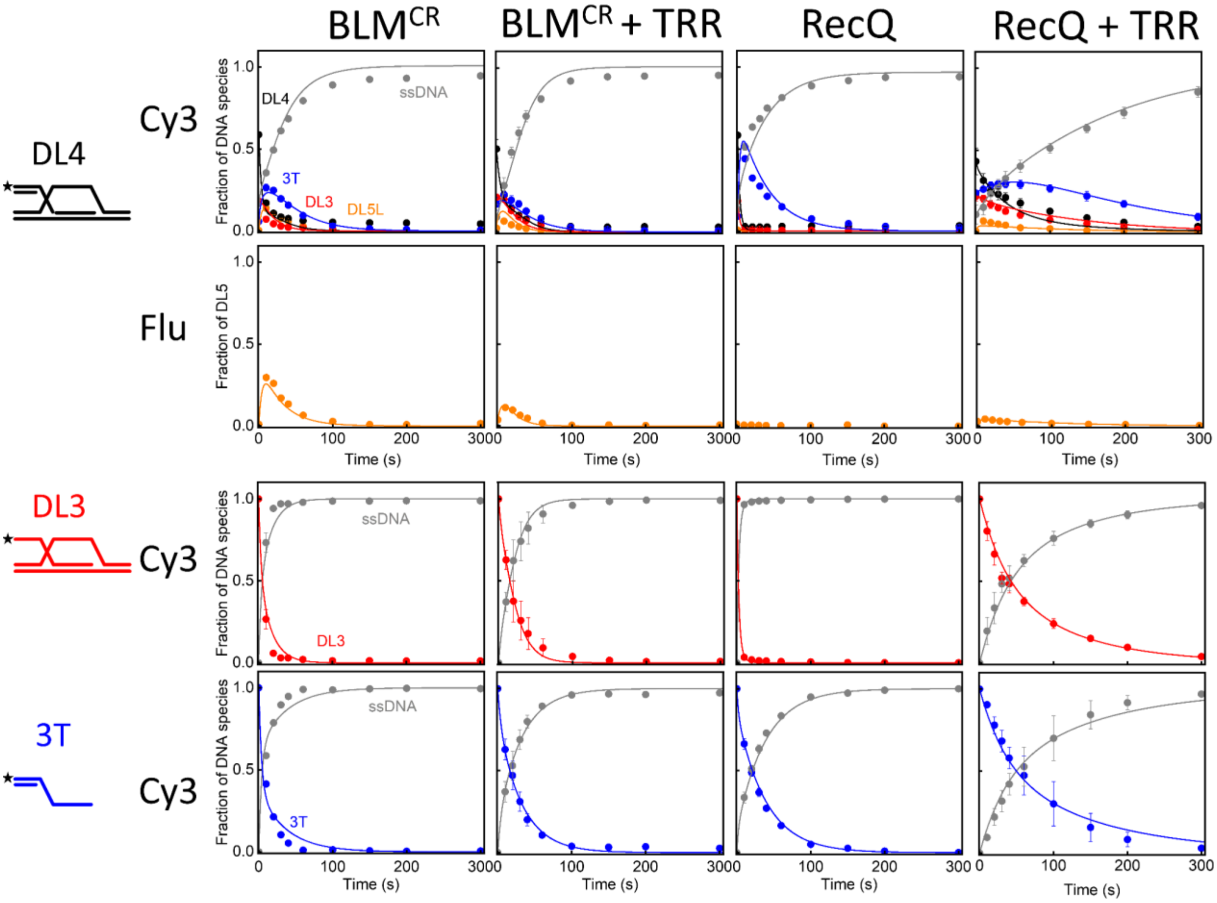

Supplementary Fig. 13. Determined fractions of DNA species from DL4 (Supplementary Fig. 11), DL3 and 3T unwinding experiments for BLM^{CR} and RecQ with or without 1.2 µM TRR.

Solid lines show global fits using the extended model shown in Supplementary Fig. 7. DNA species are color coded as in Fig. 6. For BLM^{FL} data see Fig. 8a. Determined parameters are shown in Supplementary Table 3. Means ± SEM (n = 3 for DL4 experiments, n = 2 for 3T and DL3 experiments) are shown on all panels for the detected DNA species at each time point, determined from independent experiments with individual protein constructs. Source data are provided as a Source Data file.

Figure S14

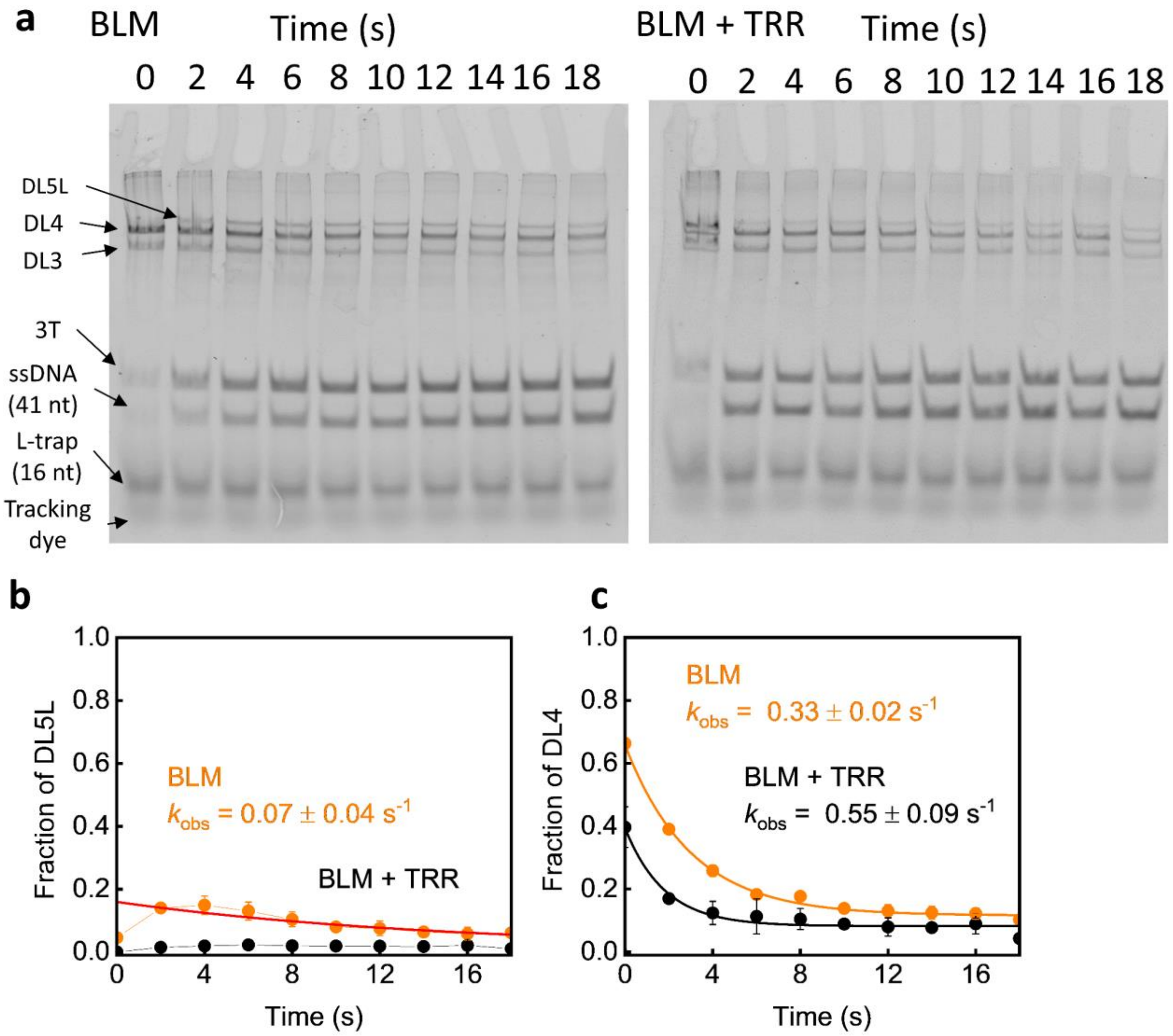

Supplementary Fig. 14. Control DL4 processing L-trap measurements performed with BLM^{FL} with or without 1.2 µM TRR at increased time resolution

(**a**) Representative Cy3 electrophoretograms from L-trap experiments performed as in Supplementary Fig. 11, but here the reaction kinetics were followed in 2-s intervals for 18 s. Experiments were independently performed twice and showed identical results**.** Reaction profiles were in line with results of Supplementary Fig. 11 and reveal that the absence of DL4L in experiments performed with 1.2 µM TRR is not caused by the increased unwinding rate of BLM and support the hypothesis that TRR increases the preference of BLM^{FL} for disruption of strand invasions (DLI and DLI' pathways on Supplementary Fig. 7).

(**b-c**) Kinetic profiles of the fractional changes of (b) DL5L (Flu signal) and (**c**) DL4 (Cy3 signal) determined from panel a. Means ± SEM for two independent experiments are shown. (b) In the absence of TRR, after initial accumulation the fraction of DL5L decreased in time as seen in Supplementary Fig. S11. In the presence of TRR only a faint signal at the position of DL5L was detectable (panel a) and its fraction increased only marginally during the experiment. Fitting of single exponential decay function to the declining phase of data obtained without TRR revealed observed rate constant of 0.07 ± 0.04 s^{-1}, close to the k_r rebinding rate (Supplementary Fig. 7) obtained via global model fitting (Supplementary Table 3). (c) The fraction of DL4 decreased in line with kinetics observed in previous experiments (Supplementary Fig. S11). Fitting of a single exponential decay function revealed observed rate constants of 0.33 ± 0.02 s^{-1} and 0.55 ± 0.09 s^{-1} for BLM^{FL} and BLM^{FL} + TRR, respectively, in line with the k_u unwinding rates determined via global fitting of the model to data in

Supplementary Fig. S11 (see also Supplementary Table 3). These results confirm that TRR enhances the D-loop unwinding activity of BLM^{FL}. Importantly, the ratio between the fraction change of DL5L and DL4 after 2 s is 0.38, indicating in a model-independent manner that BLM^{FL} maintains a balance between pathways leading to D-loop disruption and stabilization (see also Fig. 8b, Supplementary Table 3). In contrast, in the presence of TRR, DL4 is processed via other pathways, i.e., DLI and DLI', as predicted by modeling (Fig. 8b, Supplementary Table 3). Source data are provided as a Source Data file.

SUPPLEMENTARY TABLES

Supplementary Table 1: Results of global fitting of I-trap D-loop processing experiments and DNA binding K_d values

	BLM^{FL}	BLM^{CR}	BLM-dH	BLM-dWH
Apparent DNA unwinding and rebinding rate constants (s^{-1}) [a]				
k_U[b]	0.12 ± 0.03	0.14 ± 0.02	0.12 ± 0.05	0.13 ± 0.02
k_R	0.012 ± 0.005	0.019 ± 0.001	0.026 ± 0.001	0.026 ± 0.001
$k_{R'}$	0.005 ± 0.003	0.019 ± 0.02	0.016 ± 0.05	0.012 ± 0.05
Fraction of DNA binding configurations [a]				
f_{DLI}	0.45 ± 0.04	0.19 ± 0.02	0.12 ± 0.02	0.04 ± 0.01
f_{DLE}	0.02 ± 0.02	0.07 ± 0.03	0.12 ± 0.08	0.06 ± 0.01
$f_{DLI'}$	0.16 ± 0.03	0.15 ± 0.03	0.10 ± 0.07	0.11 ± 0.02
f_{DLN}	0.36 ± 0.07	0.58 ± 0.07	0.65 ± 0.07	0.78 ± 0.03
f_{3TE}	0.70 ± 0.06	0.72 ± 0.04	0.39 ± 0.03	0.26 ± 0.04
DNA binding K_d (nM)				
ss54 [c]	61 ± 10	74 ± 14	61 ± 16	520 ± 30
3T [d]	10 ± 3	24 ± 2	39 ± 3	430 ± 70
DL3 [d]	1.2 ± 0.8	< 1	< 1	56 ± 7
DL4 [d]	2.3 ± 1.3	< 1	< 1	56 ± 13

[a] Values reported were determined from global fitting of the model shown in Fig. 3 to DL4**,** DL3 and 3T unwinding data for each helicase construct (Fig. 4a). Best-fit values ± fitting standard errors (determined by the software used for fitting) are shown.

[b] Values of k_U also represent lower bounds for the rate constant of unwinding and non-productive unwinding runs.

[c] Determined from direct fluorescence anisotropy titrations using ss54-FLU (Supplementary Fig. 3a).

[d] Determined from competitive titration experiments (Supplementary Fig. 3b-e).

Supplementary Table 2: Results of global fitting of L-trap and R-trap D-loop processing experiments

	BLM^{FL}	BLM^{CR}	BLM-dH	RecQ	RecQ*	RecQ-dH
Apparent DNA unwinding and rebinding rate constants (s^{-1}) [a]						
k_U [b]	0.10 ± 0.08	0.12 ± 0.02	0.08 ± 0.06	0.12 ± 0.03	0.14 ± 0.08	0.08 ± 0.05
k_R	0.021 ± 0.013	0.019 ± 0.011	0.024 ± 0.018	0.022 ± 0.003	0.029 ± 0.018	0.012 ± 0.03
$k_{R'}$	0.003 ± 0.001	0.026 ± 0.011	0.024 ± 0.018	0.042 ± 0.019	0.029 ± 0.018	0.069 ± 0.12
Fraction of DNA binding configurations [a]						
f_{DLI}	0.42 ± 0.04	0.24 ± 0.07	0.10 ± 0.03	0.90 ± 0.05	0.73 ± 0.03	0.31 ± 0.04
f_{DLE}	0.04 ± 0.02	0.06 ± 0.03	0.04 ± 0.02	0	0	0.02 ± 0.01
$f_{DLI'}$	0.12 ± 0.06	0.15 ± 0.08	0.09 ± 0.06	0	0.03 ± 0.02	0.08 ± 0.01
f_{DLL}	0.39 ± 0.02	0.42 ± 0.02	0.39 ± 0.04	0.07 ± 0.02	0.19 ± 0.02	0.36 ± 0.02
f_{DLN}	0.06 ± 0.07	0.16 ± 0.09	0.38 ± 0.08	0.04 ± 0.04	0.04 ± 0.04	0.23 ± 0.03
f_{3TE}	0.61 ± 0.12	0.68 ± 0.13	0.32 ± 0.08	0.61 ± 0.14	0.60 ± 0.15	0.23 ± 0.06
f_{PI}	0.76 ± 0.20	0.47 ± 0.29	0.06 ± 0.05	0.87 ± 0.08	0.85 ± 0.12	0.55 ± 0.21
f_{PE}	0.11 ± 0.19	0.37 ± 0.22	0.33 ± 0.07	0.03 ± 0.02	0.05 ± 0.05	0.14 ± 0.12
f_{PN}	0.11 ± 0.11	0.14 ± 0.19	0.60 ± 0.11	0.09 ± 0.07	0.10 ± 0.11	0.30 ± 0.16

[a] Values reported were determined from global fitting of the model shown in Supplementary Fig. 7 to DL4 data from L-trap and R-trap experiments and DL3 and 3T unwinding data from I-trap experiments (Fig. 3a, RecQ unwinding data obtained from ref.[1]) for each helicase construct (Figs. 6-7).

[b] Values of k_U also represent lower bounds for the rate constant of unwinding and non-productive unwinding runs.

Best-fit values and estimation of fit robustness as error are shown. The modeling software was unable to determine fitting standard errors for most parameters. Thus, fitting uncertainty was characterized as the SD of best-fit values from 5 fitting runs starting from differentially initialized fraction ratios.

Supplementary Table 3: Results of global fitting of L-trap D-loop processing experiments in the presence of TRR

	BLM^{FL}	BLM^{FL} + TRR	BLM^{CR}	BLM^{CR} + TRR	RecQ	RecQ + TRR
Apparent DNA unwinding and rebinding rate constants (s^{-1}) [a]						
k_U [b]	0.23 ± 0.02	0.53 ± 0.07	0.27 ± 0.04	0.18 ± 0.13	0.52 ± 0.10	0.02 ± 0.01
k_R	0.07 ± 0.03	0.07 ± 0.05	0.07 ± 0.02	0.07 ± 0.03	0.10 ± 0.04	0.012 ± 0.01
$k_{R'}$	0.013 ± 0.004	0.03 ± 0.01	0.07 ± 0.01	0.07 ± 0.05	0.10 ± 0.04	0.069 ± 0.03
Fraction of DNA binding configurations [a]						
f_{DLI}	0.34 ± 0.07	0.45 ± 0.10	0.32 ± 0.10	0.25 ± 0.06	0.81 ± 0.11	0.65 ± 0.05
f_{DLE}	0.06 ± 0.03	0.06 ± 0.04	0.04 ± 0.05	0.29 ± 0.06	0.01 ± 0.01	0.09 ± 0.03
$f_{DLI'}$	0.20 ± 0.15	0.42 ± 0.12	0.12 ± 0.06	0.12 ± 0.03	0.03 ± 0.03	0.09 ± 0.01
f_{DLL}	0.32 ± 0.18	0.03 ± 0.03	0.36 ± 0.09	0.24 ± 0.09	0.05 ± 0.01	0.09 ± 0.04
f_{DLN}	0.08 ± 0.02	0.04 ± 0.02	0.15 ± 0.06	0.10 ± 0.07	0.10 ± 0.07	0.08 ± 0.03
f_{3TE}	0.43 ± 0.08	0.39 ± 0.18	0.63 ± 0.04	0.52 ± 0.35	0.23 ± 0.02	0.30 ± 0.06
f_{PI}	0.50 ± 0.12	n.d.	0.48 ± 0.07	0.47 ± 0.27	n.d.	0.77 ± 0.16
f_{PE}	0.31 ± 0.12	n.d.	0.34 ± 0.09	0.06 ± 0.01	n.d.	0.09 ± 0.05
f_{PN}	0.18 ± 0.05	n.d.	0.18 ± 0.07	0.48 ± 0.26	n.d.	0.14 ± 0.19

N.d., not determined.

[a] Values reported were determined from global fitting of the model shown in Supplementary Fig. 7 to DL4, DL3 a 3T data from L-trap experiments containing 1.2 µM TRR and BLM^{FL} or BLM^{CR} or RecQ (Fig. 8, Supplementary Fig. 11).

[b] Values of k_U also represent lower bounds for the rate constant of unwinding and non-productive unwinding runs.

Best-fit values and estimation of fit robustness as error is shown. The modeling software was unable to determine fitting standard errors for most parameters. Thus, fitting uncertainty was characterized as the SD of best-fit values from 5 fitting runs starting from differentially initialized fraction ratios.

SUPPLEMENTARY EQUATIONS

Supplementary Equations (1) and (2): Equations used for analysis of competitive fluorescence anisotropy titrations (Supplementary Fig. 3b-e) as described previously in Harami et al. [1]

In these experiments, the observed anisotropy (A) of ss54-FLU was determined by

(1) $$A = f \cdot A_{helicase.ss54} + (1-f) \cdot A_{ss54}$$

where $A_{\mathrm{helicase.ss54}}$ and A_{ss54} are the anisotropy values of the helicase.ss54-FLU complex and that of free ss54-FLU, respectively; and f is the [helicase.ss54-FLU]/[ss54-FLU]$_{\mathrm{total}}$ ratio.

According to ref. [3], the concentration of the helicase.ss54-FLU complex at a given total competitor (3T, DL3 or DL4) concentration ([competitor]$_{\mathrm{tot}}$) will thus be

(2) $$[helicase.ss54] = \frac{[ss54] \cdot \left(2 \cdot \sqrt{a^2 - 3b} \cdot \cos(\Theta/3) - a\right)}{3 \cdot K_{ss54} + \left(2 \cdot \sqrt{a^2 - 3b} \cdot \cos(\Theta/3) - a\right)}$$

where

$$a = K_{\mathrm{ss54}} + K_{\mathrm{competitor}} + [ss54]_{\mathrm{tot}} + [competitor]_{\mathrm{tot}} - [helicase]_{\mathrm{tot}},$$

$$b = K_{\mathrm{ss54}} \cdot K_{\mathrm{competitor}} + K_{\mathrm{ss54}} \cdot \left([competitor]_{\mathrm{tot}} - [helicase]_{\mathrm{tot}}\right) + K_{\mathrm{competitor}} \cdot \left([ss54]_{\mathrm{tot}} - [helicase]_{\mathrm{tot}}\right)$$

$$, \Theta = \arccos \frac{-2 \cdot a^3 + 9 \cdot a \cdot b - 27 \cdot c}{2 \cdot \sqrt{\left(a^2 - 3 \cdot b\right)^3}},$$

$$c = -[helicase]_{\mathrm{tot}} \cdot K_{\mathrm{ss54}} \cdot K_{\mathrm{competitor}},$$

K_{ss54} is the dissociation constant of the helicase-ss54-FLU interaction, and $K_{\mathrm{competitor}}$ is the dissociation constant of the helicase-competitor interaction. To reduce the number of floating parameters, A_{ss54}, $A_{\mathrm{helicase.ss54}}$ and K_{ss54} were determined in separate experimental sets and were fixed during fitting.

SUPPLEMENTARY REFERENCES

1. Harami, G. M. *et al.* Shuttling along DNA and directed processing of D-loops by RecQ helicase support quality control of homologous recombination. *Proc. Natl. Acad. Sci.* **114**, E466–E475 (2017).
2. Karow, J. K., Chakraverty, R. K. & Hickson, I. D. The Bloom's Syndrome Gene Product Is a 3'-5' DNA Helicase. *J. Biol. Chem.* **272**, 30611–30614 (1997).
3. Wang, Z. X. An exact mathematical expression for describing competitive binding of two different ligands to a protein molecule. *FEBS Lett.* **360**, 111–114 (1995).
