# Supplementary material for: The toposiomerase IIIalpha-RMI1-RMI2 complex orients human Bloom’s syndrome helicase for efficient disruption of D-loops
Source: Nat Commun. 2022 Feb 3;13:654. doi: 10.1038/s41467-022-28208-9 (PMC8813930; doi:10.1038/s41467-022-28208-9)
Supplement: Supplementary file 3 — Reporting Summary [file 41467_2022_28208_MOESM3_ESM.pdf]

nature portfolio

Corresponding author(s): Dr. Gábor Harami, Dr. Mihály Kovács

Last updated by author(s): Dec 19, 2021

# Reporting Summary

Nature Portfolio wishes to improve the reproducibility of the work that we publish. This form provides structure for consistency and transparency in reporting. For further information on Nature Portfolio policies, see our Editorial Policies and the Editorial Policy Checklist.

## Statistics

For all statistical analyses, confirm that the following items are present in the figure legend, table legend, main text, or Methods section.

| n/a | Confirmed | |
|---|---|---|
| ☐ | ☒ | The exact sample size ($n$) for each experimental group/condition, given as a discrete number and unit of measurement |
| ☐ | ☒ | A statement on whether measurements were taken from distinct samples or whether the same sample was measured repeatedly |
| ☐ | ☒ | The statistical test(s) used AND whether they are one- or two-sided<br>*Only common tests should be described solely by name; describe more complex techniques in the Methods section.* |
| ☒ | ☐ | A description of all covariates tested |
| ☒ | ☐ | A description of any assumptions or corrections, such as tests of normality and adjustment for multiple comparisons |
| ☐ | ☒ | A full description of the statistical parameters including central tendency (e.g. means) or other basic estimates (e.g. regression coefficient) AND variation (e.g. standard deviation) or associated estimates of uncertainty (e.g. confidence intervals) |
| ☐ | ☒ | For null hypothesis testing, the test statistic (e.g. $F$, $t$, $r$) with confidence intervals, effect sizes, degrees of freedom and $P$ value noted<br>*Give P values as exact values whenever suitable.* |
| ☒ | ☐ | For Bayesian analysis, information on the choice of priors and Markov chain Monte Carlo settings |
| ☒ | ☐ | For hierarchical and complex designs, identification of the appropriate level for tests and full reporting of outcomes |
| ☒ | ☐ | Estimates of effect sizes (e.g. Cohen's $d$, Pearson's $r$), indicating how they were calculated |

*Our web collection on statistics for biologists contains articles on many of the points above.*

## Software and code

Policy information about availability of computer code

| | |
|---|---|
| Data collection | Fluorescence anisotropy: inbuilt software for Synergy H4<br>Hybrid Multi-Mode Microplate Reader (BioTek); fluorescent image scanning: inbuilt software for Typhoon TRIO+ Variable Mode Imager (Amersham Biosciences) |
| Data analysis | Data representation and fitting: OriginLab 8.0 (Microcal corp.), Pixel densitometry: GelQuant Pro v12 (DNR Bio Imaging Ltd.), Kinetic modeling: KinTek Global Kinetic Explorer 4.0 (KinTek). (corresponding references included in the manuscript) |

For manuscripts utilizing custom algorithms or software that are central to the research but not yet described in published literature, software must be made available to editors and reviewers. We strongly encourage code deposition in a community repository (e.g. GitHub). See the Nature Portfolio guidelines for submitting code & software for further information.

## Data

Policy information about availability of data

All manuscripts must include a data availability statement. This statement should provide the following information, where applicable:

- Accession codes, unique identifiers, or web links for publicly available datasets
- A description of any restrictions on data availability
- For clinical datasets or third party data, please ensure that the statement adheres to our policy

All data generated or analysed during this study are included in this published article and its Supplementary Information files. The pdb file 4O3M was used for the presentation of the BLM 3D structure was obtained from the Protein Data Bank (https://www.rcsb.org/structure/4O3M). Source data are provided with this paper.

# Field-specific reporting

Please select the one below that is the best fit for your research. If you are not sure, read the appropriate sections before making your selection.

☒ Life sciences ☐ Behavioural & social sciences ☐ Ecological, evolutionary & environmental sciences

For a reference copy of the document with all sections, see nature.com/documents/nr-reporting-summary-flat.pdf

# Life sciences study design

All studies must disclose on these points even when the disclosure is negative.

| | |
|---|---|
| Sample size | We have not predetermined the sample sizes based on statistical methods, instead we chosed sample sizes based on our previous results (Harami et. al. 2017 PNAS). Generally for experiments relevant for the main conclusions we aimed for 3 independent experiments with various protein contsructs. These are typical repetition numbers for in vitro enzyme activity studies. Sample sizes are reported in the figure legends. |
| Data exclusions | No data were excluded from this study. |
| Replication | Sample sizes are reported for all measurements in Figure legends and Methods. Conclusions were confirmed with different protein preparations. |
| Randomization | There was no experimental group allocations for this study. The activities of our protein variants were measured in paralel in a random order by multiple lab members. |
| Blinding | Blinding was not relevant for our in vitro studies as there was no experimental group allocations and biophysical parameters were quantitatively assessed along with controls. |

# Reporting for specific materials, systems and methods

We require information from authors about some types of materials, experimental systems and methods used in many studies. Here, indicate whether each material, system or method listed is relevant to your study. If you are not sure if a list item applies to your research, read the appropriate section before selecting a response.

## Materials & experimental systems

| n/a | Involved in the study | |
|---|---|---|
| ☒ | ☐ | Antibodies |
| ☒ | ☐ | Eukaryotic cell lines |
| ☒ | ☐ | Palaeontology and archaeology |
| ☒ | ☐ | Animals and other organisms |
| ☒ | ☐ | Human research participants |
| ☒ | ☐ | Clinical data |
| ☒ | ☐ | Dual use research of concern |

## Methods

| n/a | Involved in the study | |
|---|---|---|
| ☒ | ☐ | ChIP-seq |
| ☒ | ☐ | Flow cytometry |
| ☒ | ☐ | MRI-based neuroimaging |
